# Promoting deceased organ and tissue donation registration in family physician waiting rooms (RegisterNow-1): a pragmatic stepped-wedge, cluster randomized controlled registry trial

**DOI:** 10.1186/s12916-022-02266-8

**Published:** 2022-03-03

**Authors:** Alvin Ho-ting Li, Amit X. Garg, Jeremy M. Grimshaw, Versha Prakash, Alexie J. Dunnett, Stephanie N. Dixon, Monica Taljaard, Joanna Mitchell, Kyla L. Naylor, Cathy Faulds, Rachel Bevan, Leah Getchell, Greg Knoll, S. Joseph Kim, Jessica Sontrop, Allison Tong, Lise M. Bjerre, Karyn Hyjek, Donna Currie, Susan Edwards, Mike Sullivan, Linda Harvey-Rioux, Justin Presseau

**Affiliations:** 1 Clinical Epidemiology Program, Ottawa Hospital Research Institute, Ottawa, ON Canada; 2 Lawson Health Research Institute, London, ON Canada; 3 Institute for Clinical Evaluative Sciences (ICES) Western, London, ON Canada; 4 Department of Epidemiology and Biostatistics, Western University, London, ON Canada; 5 Division of Nephrology, Western University, London, ON Canada; 6 Department of Medicine, University of Ottawa, Ottawa, ON Canada; 7 Trillium Gift of Life Network, Toronto, ON Canada; 8 School of Epidemiology and Public Health, University of Ottawa, Ottawa, ON Canada; 9 Citizen partner, London, ON Canada; 10 Department of Family Medicine, Western University, London, ON Canada; 11 University Health Network, University of Toronto, Toronto, ON Canada; 12 Sydney School of Public Health, University of Sydney, Sydney, NSW Australia; 13 Department of Family Medicine, University of Ottawa, Ottawa, ON Canada; 14 School of Psychology, University of Ottawa, Ottawa, ON Canada

**Keywords:** Stepped-wedge trial, Cluster randomized trial, Pragmatic trial, Behaviour change techniques, Organ donation, Organ registration, Family physician offices

## Abstract

**Background:**

The shortage of available organs for life-saving transplants persists worldwide. While a majority support donating their organs or tissue when they die, many have not registered their wish to do so. When registered, next of kin are much more likely to follow-through with the decision to donate. In many countries, most people visit their family physician office each year and this setting is a promising, yet underused, site where more people could register for deceased organ donation. Our primary aim was to evaluate the effectiveness of an intervention to promote organ donation registration in family physician’s offices.

**Methods:**

We developed an intervention to address barriers and enablers to organ donation registration that involved physician office reception staff inviting patients to register on a tablet in the waiting room while they waited for their appointment. We conducted a cross-sectional stepped-wedge cluster randomized controlled registry trial to evaluate the intervention. We recruited six family physician offices in Canada. All offices began with usual care and then every two weeks, one office (randomly assigned) started the intervention until all offices delivered the intervention. The primary outcome was registration for deceased organ donation in the provincial organ registration registry, assessed within the 7 days of the physician visit. At the end of the trial, we also conducted interviews with clinic staff to assess any barriers and enablers to delivering the intervention.

**Results:**

The trial involved 24,616 patient visits by 13,562 unique patients: 12,484 visits in the intervention period and 12,132 in the control period. There was no statistically significant difference in the percentage of patients registered for deceased organ donation in the intervention versus control period (48.0% vs 46.2%; absolute difference after accounting for the secular trend: 0.12%; 95% CI: − 2.30, 2.54; *p*=0.92). Interviews with clinic staff indicated location of the tablet within a waiting room, patient rapport, existing registration, confidence and motivation to deliver the intervention and competing priorities as barriers and enablers to delivery.

**Conclusions:**

Our intervention did not increase donor registration. Nonetheless, family physician offices may still remain a promising setting to develop and evaluate better interventions to increase organ donation registration.

**Trial registration:**

NCT03213171

**Supplementary Information:**

The online version contains supplementary material available at 10.1186/s12916-022-02266-8.

## Background

There is a persistent shortage of organs available for transplant worldwide. In 2019, over 4350 people in Canada were on an organ donor waiting list, and over 223 people died while waiting [1].

Individuals in many countries can register their decision (opt-in) to donate their organs and tissues when they die [2]. At the time of death, information from the registry is then provided to the next-of-kin to support the donation decision on behalf of their loved one. In Ontario (Canada), knowing a loved one’s wishes makes a difference: 90% of families consent when the deceased registered for organ donation, compared to 50% when the deceased is not registered [3]. Public opinion polling suggests that more than 90% of Canadians are in favour of organ donation [4]. However, this positive support is not reflected in actual registration rates. In Ontario, only 35% of eligible citizens (those 16 years of age or older) are registered for organ and tissue donation in the provincial donor registry [5]. Similar to several other jurisdictions, one way citizens are made aware of the opportunity to register for deceased organ donation is when they visit a government office to obtain or renew a driver’s motor vehicle license [2, 6]. While an important part of an overall registration strategy, new supplemental opportunities are needed to register in different settings with similar widespread, population-level reach.

Family physician offices are a promising—yet under-investigated—setting for promoting organ donation registration [7, 8]. Family physicians are a trusted source of health information and most Canadians see their family doctor at least once a year. In the UK, new patients can also register for organ donation with their general practitioner [9]. Family physician offices represent a setting in which citizens are in a health-focused mindset.

A systematic review of nine studies concluded that family physician office-based interventions may increase donor registration; however, more rigorously designed and adequately powered studies are needed [7]. We developed an intervention (RegisterNow-1) to leverage the opportunity in family physician waiting rooms to encourage organ donation registration [10] and conducted a stepped-wedge cluster randomized trial. The aim of this study was to evaluate whether our intervention increased the number of patients registering for organ donation.

## Methods

### Trial design

**Fig. 1:**
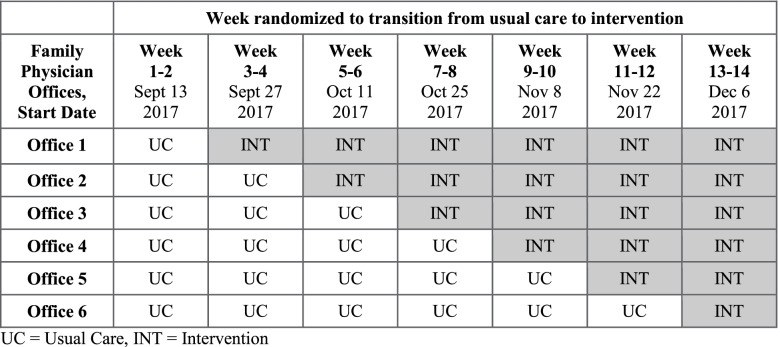
Diagram of the stepped-wedge design

We used the Consolidated Standards of Reporting Trials (CONSORT) statement extension for stepped-wedge cluster randomized trials in the reporting of the trial (Additional File 1: Table S1) [11, 12]. We have previously published a detailed study protocol [10]. There were no major deviations from the protocol. Briefly, we conducted a 14-week pragmatic, provincial registry-based, stepped-wedge cluster randomized controlled trial to evaluate the effects of an intervention to promote organ donor registration compared to no intervention (usual care) in six family physician offices in Southwestern Ontario (Canada) from September to December 2017 (see Fig. 1 for a diagram of the stepped-wedge design). Cluster randomization was required as the intervention was delivered at the level of the practice. A stepped-wedge roll-out was chosen because we suspected it is unlikely that family physicians would agree to be randomized unless they were guaranteed, at some stage during the trial, to receive the intervention.

The ethics boards at Western University and the Ottawa Health Science Network Research Ethics Board approved the trial. We sought and received a waiver of the need for consent for data collection because we did not require the collection of any identifiable private information from patients and we obtained a waiver for the need for consent for the study intervention because it was deemed that our intervention posed minimal to no risk to patients (OHSN: 20170236-1H and Western 109297). We obtained consent from family physicians participating in enrolled sites to link their physician identifier to the databases at the Institute for Clinical Evaluative Sciences (ICES). These datasets were linked using unique encoded identifiers and analysed at ICES. ICES is an independent, non-profit research institute funded by an annual grant from the Ontario Ministry of Health (MOH) and the Ministry of Long-Term Care (MLTC). As a prescribed entity under Ontario’s privacy legislation, ICES is authorized to collect and use health care data for the purposes of health system analysis, evaluation and decision support. Secure access to these data is governed by policies and procedures that are approved by the Information and Privacy Commissioner of Ontario. The use of data in this project was authorized under section 45 of Ontario’s Personal Health Information Protection Act, which does not require review by a Research Ethics Board.

### Patient/public involvement

Five members of the public (JM, DC, SE, MS, LH-R) formed our citizen panel and were involved in all aspects of the design and conduct of the research, including the grant application (Additional File 1: Table S2). One of the key components of our intervention involved asking reception staff to provide a pamphlet that was designed to include content that addressed previously-identified barriers and enablers to organ donor registration to patients upon check-in. During the design stage, our citizen partners actively contributed to and reviewed the contents of the pamphlet. The final pamphlet contained personal stories as transplant recipients from the citizen panel, step-by-step instructions on registering and asking patients to confirm if they are indeed registering. The citizen panel reflected on their experiences visiting their family physician to help mitigate potential issues that could arise in delivering the intervention. They were also invited to comment and provide feedback on the manuscript.

### Participants

We included family practices that saw at least 100 patients/week. For reasons of convenience, we restricted our recruitment to practices in Southwestern Ontario. As this was a pragmatic registry trial, we had few eligibility criteria for patients to ensure practicality for participating sites: we asked family practices to deliver our intervention during their assigned trial period to all patients aged ≥ 16 years with a valid Ontario health card who visited the office as an outpatient.

### Intervention description

#### Intervention arm

Ontario operates a donor registry within which any citizen aged 16 or older can register their decision to become a deceased organ and tissue donor. Registration can be completed in person at a Service Ontario centre, by mailing a registration form, or online at beadonor.ca. Donor status is then updated and appears on the back of a patient’s health card upon renewal. We focused on the beadonor.ca website for enabling registration in this intervention. The intervention was designed to be practical for family physician offices by involving reception staff and Internet-enabled tablets and leveraging wait-time in waiting rooms to encourage donation registration. Our trial protocol describes the methods used for developing our intervention [10]. We specified the intervention components using the Theoretical Domains Framework (to describe the targeted barriers and enablers to organ donation registration), the Behaviour Change Techniques (BCT) Taxonomy v1 (to describe the strategies used to address each barrier) and the TIDIER checklist (see Table 2 in the protocol for full description) [13, 14]. Briefly, our intervention consisted of three components: (1) reception staff identifying patients who had not yet registered for organ donation by checking the back of every patient’s health card for the donor status indicator; (2) reception staff providing pamphlets designed to address previously identified barriers and enablers to organ donor registration; and (3) providing an Internet-enabled tablet in the waiting rooms for secure online registration (see Table 1). Reception staff received standardized training to clarify the intervention, trial logistics, and to practice delivering the intervention using role-playing and problem solving how to respond to anticipated questions or issues that could be raised by patients in the 2–4 weeks prior to the allocated start date at each site.

**Table 1:** Intervention components, how delivered and underlying behaviour change techniques designed to be delivered

Intervention component	How delivered	Behaviour Change Techniques
Case finding	Reception staff identified patients who had not yet registered for organ donation by checking the back of every patient’s health card for their donor status	4 BCTs: instruction on how to perform the behaviour; social support [practical]; prompt/cues; information about others’ approval
Address previously identified barriers and enablers to organ donor registration	Reception staff provided pamphlets	10 BCTs: instruction on how to perform the behaviour; information about others’ approval; credible source; social comparison; prompts/cues; verbal persuasion of capability; vicarious consequences; information about social and environmental consequences; salience of consequences; information about emotional consequences
Immediate and available opportunity to register	An Internet-enabled tablet in the waiting rooms	2 BCTs: adding objects to the environment; prompts/cues

*Note*. BCTs described using labels proposed by the Behaviour Change Techniques (BCT) Taxonomy v1

#### Control arm

Our control condition consisted of usual care until the practice was randomized to begin delivering the intervention (pamphlets, iPads and iPad stands were only delivered to practices at the start of their randomized intervention period). Consistent with the stepped-wedge trial design, all practices began in usual care and eventually crossed over to deliver the intervention at their randomly assigned trial period.

### Outcomes

Our pre-specified primary outcome was the prevalence of all patients aged ≥16 years who visited the family practice office during each 2-week interval and were registered for deceased organ donation within 7 days following the family physician visit (to allow patients the time to discuss with family), as captured in the Ontario organ donor registry. However, we designed the intervention so that most registrations could take place immediately in the waiting rooms. Pre-specified secondary outcomes were registration within 1 day, 14 days and 30 days of the physician visit.

### Planned subgroup analyses

We pre-specified subgroup analyses to examine any differential effect of our intervention by age [younger (≤40 years) vs. older (>40 years)] and sex (male vs. female).

### Sample size

A priori, we calculated our sample size based on detecting an absolute increase of 10% in the prevalence of registrations which was considered both a realistic and clinically important difference. Six practices with an average of 250 patients attending each practice every 2 weeks had 80% power to detect a 10% absolute increase of donor registration, assuming a control arm proportion of 0.50, an intraclass correlation of 0.06, and cluster autocorrelation coefficient of 0.80 using a two-sided test at the 5% level of significance (see protocol for more details on sample size calculation assumptions and for a sensitivity analyses that varied parameters) [10]. We did not account for the possibility of multiple visits by individual patients due to the anticipated rarity of repeat visits over the short study duration.

### Data collection methods

We obtained baseline and outcome information from multiple linked healthcare administrative databases housed at ICES*.* We brought in a study-specific dataset consisting of our list of family physicians consenting to participate in order to reliably determine which patients visited the recruited family physicians and their visit dates. We used the Registered Persons Database, the Canadian Institute for National Information (CIHI) - National Ambulatory Care Reporting System (NACRS), CIHI – Discharge Abstract Database (DAD), Ontario Health Insurance Plan (OHIP) database and the ICES Physician Database to obtain personal demographics and health characteristics. We also used the OHIP database and physician billing records to the government to establish a time-stamp of the date of patient visits, and Ontario’s Organ Donor Registry (ODR) to obtain our outcome measurement of organ donor registration status.

### Randomization, blinding and inclusion criteria

A statistician (MT) not involved in intervention delivery used computer-generated random numbers to allocate sites to different start dates. No covariates were used in the allocation. Sites were randomized by the trial statistician in one batch at the beginning of the trial, but the timing of the transition from control to intervention for each site was only revealed to the research team 2–4 weeks prior to the transition date. At that point, the research team revealed the start date to the next upcoming site and proceeded with booking staff training.

We used the above-described routinely collected administrative data at ICES to identify eligible trial participants. The trial included all patients ≥16 years of age that visited the six family physician offices in the trial over a 14-week period. Patient visits including the date of the visit were identified by family physician billing claims.

### Statistical methods

We used descriptive statistics to compare site and patient demographic characteristics between the control and intervention conditions. All analyses were conducted by intention-to-treat. We analysed our primary and secondary outcomes at the visit-level using mixed-effects regression, including intervention status and categorical time as fixed effects with a random intercept and time effect defined at the level of the family physician office [15, 16]. The inclusion of the two random effects accounted for the within-period and between-period intra-cluster correlations [15]. Additionally, we used the Kenward and Roger method to correct for the potential inflation of type I error rate due to the small number of clusters [17]. To express the estimated effect of our intervention as an absolute difference, we used the binomial distribution and an identity link function and reported the corresponding 95% confidence intervals. We adjusted for three pre-specified patient-level covariates that were previously shown to be associated with donor registration: age, sex, and neighbourhood income quintile (according to fifths of average neighbourhood income) [18]. Missing data for income quintile (<1%) was imputed using the mode (income quintile of 3). We conducted subgroup analyses by including an interaction between the subgroup variable and both the treatment and period indicator. We conducted all analyses using SAS v9.4 and statistical significance was assessed at the 5% level.

### Post-trial process evaluation

We conducted a qualitative study alongside the trial using semi-structured interviews to understand enablers and barriers to delivering the intervention by routine clinic reception staff. This study was conducted two months after completion of the trial, prior to the trial results being analysed. The interview guide was informed by the Theoretical Domains Framework [19, 20]. A member of the research team (AD) contacted a nominated reception staff member at each clinic via email with information regarding the qualitative evaluation. A $10 gift card was provided to each participant. All interviews were audio-recorded and transcribed, and analysis involved directed content analysis of barriers and enablers into Theoretical Domains Framework domains followed by thematic analysis of barriers and enablers within domains. In addition, we assessed the fidelity of delivering the intervention by tracking how many pamphlets were delivered in each practice by collecting the residual pamphlets at the end of the trial period.

## Results

### Recruitment and participants

**Fig. 2:**
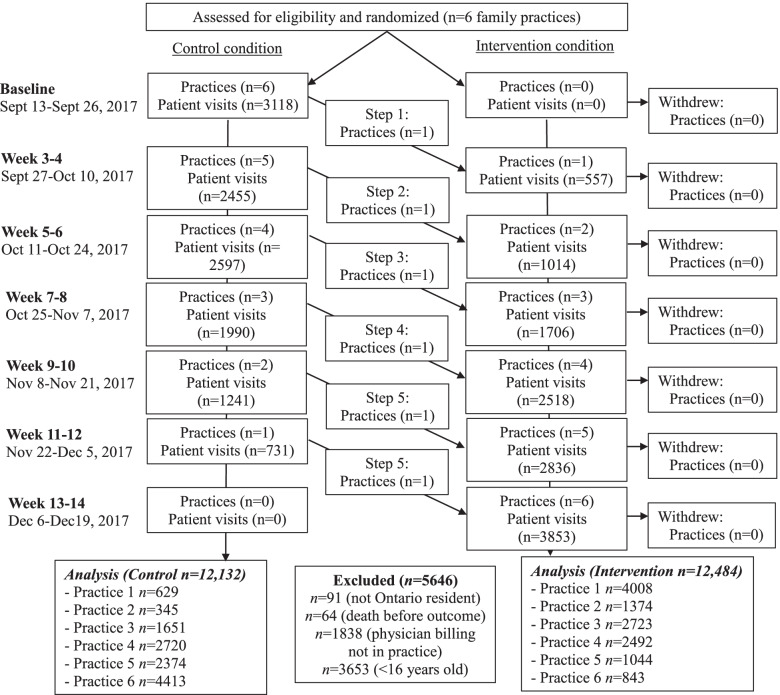
Flow chart of study participants

We recruited six family physician offices; the number of physicians ranged from 2 to 4 at each site. We obtained consent from all physicians (*n*=25) to link their physician identifiers to the provincial administrative databases for patient descriptors and outcome assessment. Over the course of the 14-week trial from September to December 2017, there were *n*=12,484 patient visits to a physician during the intervention period and *n*=12,132 patient visits during the control period (see Fig. 2 for study participant flowchart). Based on our pre-specified exclusion criteria, we excluded 91 non-Ontario residents, 64 for data cleaning (i.e., death before the index date) and 1838 whose location of billing was not in a clinic. We further excluded 3653 records, which were associated with patient visits for individuals less than 16 (since patients less than 16 years of age cannot register for organ donation in Ontario). See Table 2 for baseline demographics for intervention and control periods, indicating broad similarity.

**Table 2:** Baseline Characteristics of trial participants

Characteristics	Control (***n***=12,132)	Intervention (***n***=12,484)
Median age (25th, 75th percentile)	55 (37–69)	59 (41–72)
Age category, %		
16 to 29 years	1734 (14%)	1599 (13%)
30 to 40 years	1768 (15%)	1488 (12%)
41 to 65 years	4775 (39%)	4647 (37%)
66 to 80 years	2774 (23%)	3223 (26%)
80+ years	1081 (9%)	1527 (12%)
Female, %	7696 (63%)	7976 (64%)
Rural, %	1052 (9%)	2027 (16%)
Neighbourhood income quintile^a^, %		
1 (lowest quintile)	2365 (20%)	1789 (14%)
2	2349 (19%)	2119 (17%)
3	2174 (18%)	2317 (19%)
4	2456 (20%)	3143 (25%)
5 (highest quintile)	2743 (23%)	3077 (25%)
Comorbid conditions, %		
Diabetes	2084 (17%)	2071 (17%)
Cancer	3119 (26%)	3436 (28%)
Congestive heart failure	670 (6%)	834 (7%)
Chronic kidney disease	249 (2%)	288 (2%)
Chronic liver disease	528 (4%)	493 (4%)
Chronic lung disease	2594 (21%)	2540 (20%)
Median family pPhysician visits in the past year, #, (Q1–Q3)	5 (2–9)	5 (3–9)

Q1: lower quartile, Q3: upper quartile

^a^Less than 1% missing data

### Primary trial outcome

The percentage of patients registered for organ/tissue donation within 7 days of visit was 46.2% in the control period vs. 48.0% in the intervention period. Accounting for the study design and adjusting for the pre-specified covariates this difference was not statistically significant (adjusted mean difference: 0.12%; 95%CI: − 2.30 to 2.54; *p*=0.92). The within period ICC was 0.004, and cluster autocorrelation coefficient was 0.859 (Additional File 1: Table S3).

Contrary to our expectations, only 60% of our cohort had a single visit. We attempted to account for correlation in multiple visits by the same patient by including a random intercept for the patient, however, this model failed to converge. We then re-analysed the data by including a single, randomly selected visit for patients with multiple visits, thereby removing the correlation at the individual level. Our results remained unchanged: an adjusted mean difference of 0.12% (− 2.30 to 2.54%) *p* value = 0.9238.

### Adverse events

There were no reported adverse events. However, ≤5 registrants (≤ 0.0003% of patients in our study) that were registered at baseline in both intervention and control period did remove their registration for organ and tissue donation following the family physician visit.

### Secondary trial outcomes

There was no significant difference between the control and intervention period on donor registration within 1 day of the physician visit (46.2% vs 47.9%; adjusted mean difference: 0.12%; 95%CI: − 2.33, 2.56;), within 14 days (46.3% vs 48.1%; adjusted mean difference; adjusted mean difference: 0.25%; 95%CI: − 2.21, 2.71) or within 30 days (46.4% vs 48.2%; adjusted mean difference: − 0.1%; 95%CI: − 2.28, 2.08).

### Subgroup analyses

There were no significant differences between intervention and control periods in any of our pre-specified subgroups (Additional File 1: Table S4).

### Post-trial process evaluation results

We delivered a total of 2250 pamphlets to all sites. At the end, 1308 pamphlets remained. This suggests that reception staff delivered pamphlets to up to 942 out of the 12,484 patients (7.6%) visiting during the intervention period.

### Barriers and enablers to intervention delivery interviews

One physician and 14 reception staff (at least one from each site) were interviewed. Most were women and the mean number of years in their job reported was 6.5 years (range: 8 months to 34 years). Interviews lasted between 9 to 29 minutes (mean=17). We identified five themes that reflected the barriers and enablers to delivering the intervention:

- *Theme 1: The tablet, its placement as a visual cue and website user-friendliness (Environmental Context and Resources).* Healthcare staff reported that the Internet-enabled tablet (iPad) placed in the waiting room acted as both an enabler and a barrier. It was seen as an enabler as reception staff reported that witnessing patients register for organ donation in the waiting room motivated them to continue to prompt additional patients. The tablets also served as a reminder for some reception staff, particularly when placement was in their line of vision. While placement of the tablet at each site was informed by waiting room practicalities and staff preferences, future studies could better ensure a direct line of sight for reception staff if possible. It was also seen as a barrier, with staff reporting that the central registration website was not as user-friendly as hoped, especially among older patients.
- *Theme 2: Caution and sensitivity to harness rapport in delivering the intervention (Beliefs about consequences, Social influences, Emotion, Professional Role and Identity) S*ome reported being selective about to whom they delivered the intervention. Reception staff felt that they did not want to bother patients with an acute illness regarding organ donor registration, and intervention training emphasized that they should use their best judgement for a given patient. Reception staff were optimistic and motivated during training for delivering the intervention, but during post-trial interviews, some staff reported being worried about the sensitivity of the topic and about causing worry or distress especially when they did not know the reason for a patient visit. Such experiences led to some feeling as if it was not always their role to promote organ donation because “[they] don’t see what’s happening with the actual person with their health”. Some reception staff recommended that it may be more appropriate for nurses and physicians who have access to patient charts to bring up organ donation. Nevertheless, some reception staff thought that their existing relationship with their patients was a facilitator to discussing organ donation, while other staff did not wish to harm their trust. Several reception staff reported it was “uncomfortable” and “awkward” to discuss organ donation with patients.
- *Theme 3: Competing priorities (Environmental Context and Resources).* Our intervention occurred as the same time as the flu and holiday season (September to December). Reception staff thought that this was a particularly busy time of the year for them, which may have prevented them from fully delivering the intervention.
- *Theme 4: Already registered (Reinforcement).* Some mentioned a greater proportion of patients were already registered for organ donation than they expected, which demotivated them from delivering the intervention.
- *Theme 5: Confidence and motivation to deliver the intervention (motivation; beliefs about capabilities).* Many reception staff noted that the training sessions provided helped to improve their confidence in delivering the intervention and that they were motivated to participate. However, a participant from a site that was randomized to an earlier start date noted that the length of intervention was too long and recommended that a week-long intervention may have been preferred.

## Discussion

To our knowledge, this is the first pragmatic Canadian trial testing the effectiveness of an intervention to increase donor registration in family physician’s offices. Our trial showed that the intervention had no clinically important effect on increasing registration for deceased organ donation. Our process evaluation identified several barriers that hindered the delivery of the intervention as intended, and points to opportunities for refinement for future iterations. Barriers and enablers highlighted by staff included the location of the tablet within a given waiting room, balancing patient rapport with asking about registration, patients already being registered for organ donation, confidence and motivation to deliver the intervention and competing priorities.

### Comparison with other studies

There are important differences between our trial and others, especially trials that reported a beneficial intervention effect. First, other trials tend to enrol volunteer patients who by their nature are already interested in registering for organ donation; whereas our trial was deliberately pragmatic and representative of how patients might typically be approached in routine settings [7, 21, 22]. For example, a USA-based study assessed the effects of an intervention which consisted of a video addressing concerns on organ donation consent and then prompted patients to choose a question regarding organ donation barriers to discuss with their primary care provider [21]. In this trial, 21% of patients assessed for eligibility declined to participate and were thus excluded, whereas the pragmatic nature of our trial was such that every eligible patient in our trial was included. Second, our intervention was not delivered by research staff or health care professionals but rather reception staff, designed to be more easily distributed and scaled if effective. Third, while many patients were already registered for organ donation (~41%; consistent with provincial rates), over half had not yet registered.

In a recent trial based in primary care, Degenholtz and colleagues used a standard two-arm trial design where the intervention arm involved primary care staff providing a physical (paper) donor designation form directly to patients to fill out [8]. They showed that 761 (8.1%) of the 9428 people who were not already registered completed the designation form to be organ donors and none in the comparatively smaller control group who were not directly provided with a form registered (0%; 0/579).

### Strengths and limitations

Our study had several strengths. We objectively assessed organ donor registration behaviour through routinely collected data by linking provincial registry data to family physician billing data. Using administrative data provided an objective measure of donor registration compared to many previous studies that assessed self-reported intention (which does not necessarily lead to increased registration) or self-reported behaviour (which may be subject to social desirability bias) [22]. It also demonstrates the capacity to leverage routinely collected data for a rigorous and pragmatic evaluation of interventions that do not add an undue burden to family practices or patients.

We used a pragmatic approach with broad eligibility criteria that would be representative of how such an intervention could be rolled out in practice. Further, we tested a feasible, scalable intervention co-developed with patient and citizen partners and the provincial organ procurement agency that was delivered by reception staff rather than research staff.

While this intervention did not show a clinically important effect, the demonstrated potential of leveraging the links between physician billing (as an indicator of date of visit) to the provincial organ donor registry highlights real opportunities for iterating on the approach reported herein. This approach could also be used to evaluate interventions focused on promoting other health behaviours in primary care.

Limitations to study generalizability included having a small number of clusters in a single geographical area. Further, we did not measure the number of patients that were correctly identified by the reception staff and received the intervention. We were unable to assess the donor registration rates of persons accompanying patients.

### Future studies

The family physician office remains a promising setting to develop and test interventions that can improve organ donor registration. We encountered some delivery issues that were reflected in our process evaluation of barriers experienced by reception staff. In principle, many of these issues could be addressed in a future iteration of the intervention to promote greater fidelity of delivery. For instance, enhanced training of reception staff could emphasize that more patients than they might expect may already be registered but this should not dissuade them from identifying those who have not. While primary care is a busy setting, aiming to deliver the intervention at a time less prone to competing priorities such as flu season may enhance delivery, as would selecting a particularly opportune time of the year (e.g., accompanying a public campaign for organ donor month). Aligning with organ donor month or any other mass public awareness campaign may help to reassure and ‘give permission’ to reception staff to approach patients. Exploring the potential and feasibility of involving other primary care professionals in directly prompting organ donation registration, including physicians, nurses and healthcare assistants (e.g. Penn-Jones 2020 [9]) is an important area for further research to identify a feasible, acceptable, and scalable solution. Given the potential of primary care relative to other settings in which organ donation registration is typically promoted, further development and testing of intervention in family practice seems warranted. Any such future studies should endeavour to use objective measures of donor registration behaviour and leverage rigorous trial designs to evaluate such interventions [22].

## Conclusion

RegisterNow-1 had no significant effect on donor registration. A different approach is needed to realize the potential of increasing organ donor registration in family physician offices.

## Supplementary Information

**Additional file 1.**

## Data Availability

The dataset from this study is held securely in coded form at ICES. While legal data sharing agreements between ICES and data providers (e.g., healthcare organizations and government) prohibit ICES from making the dataset publicly available, access may be granted to those who meet pre-specified criteria for confidential access, available at www.ices.on.ca/DAS (email: das@ices.on.ca). The full dataset creation plan and underlying analytic code are available from the authors upon request, understanding that the computer programs may rely upon coding templates or macros that are unique to ICES and are therefore either inaccessible or may require modification.

## Abbreviations

BCT: Behaviour Change Techniques
CIHI: The Canadian Institute for National Information
DAD: CIHI – Discharge Abstract Database
ICES: Institute for Clinical Evaluative Sciences
INT: Intervention
MLTC: The Ministry of Long-Term Care
MOH: Ontario Ministry of Health
NACRS: National Ambulatory Care Reporting System
OHIP: Ontario Health Insurance Plan
SPOR: Strategy for Patient Oriented Research
UC: Usual care
