# Supplementary material for: Promoting deceased organ and tissue donation registration in family physician waiting rooms (RegisterNow-1): a pragmatic stepped-wedge, cluster randomized controlled registry trial
Source: BMC Med. 2022 Mar 3;20:75. doi: 10.1186/s12916-022-02266-8 (PMC8892727; doi:10.1186/s12916-022-02266-8)
Supplement: Supplementary file 1 — Additional file 1. [file 12916_2022_2266_MOESM1_ESM.docx]

**Supplemental Tables**

Table S1: Checklist of information to include when reporting a stepped wedge cluster randomized trial (SW-CRT) for RegisterNow-1

Table S2: GRIPP2 Checklist for Patient Involvement

Table S3: Within period and Between Period ICC for donor registration at 1 day, 7 days, 14 days and 30 days

Table S4: Impact of the Intervention on 7-day Donor Registration (primary outcome) by including an interaction between the subgroup variables (male vs. female, age < 40 vs, age ≥ 40) and both the treatment and period indicator

Table S1: Checklist of information to include when reporting a stepped wedge cluster randomized trial (SW-CRT) for RegisterNow-1

| Topic | Item no | Checklist item | Page no |
| --- | --- | --- | --- |
| Title and abstract | | | |
|  | 1a | Identification as a stepped wedge cluster randomised trial in the title. | 1 |
|  | 1b | Structured summary of trial design, methods, results, and conclusions (see separate SW-CRT checklist for abstracts). | 3 |
| Introduction | | | |
| Background and objectives | 2a | Scientific background. Rationale for using a cluster design and rationale for using a stepped wedge design. | 4-5 |
|  | 2b | Specific objectives or hypotheses. | 4 |
| Methods | | | |
| Trial design | 3a | Description and diagram of trial design including definition of cluster, number of sequences, number of clusters randomised to each sequence, number of periods, duration of time between each step, and whether the participants assessed in different periods are the same people, different people, or a mixture. | 4-5; Figure 1; See also published protocol (Li et al, 2017, *Trials*) |
|  | 3b | Important changes to methods after trial commencement (such as eligibility criteria), with reasons. | n/a (stated on p 4) |
| Participants | 4a | Eligibility criteria for clusters and participants. | 5  See also published protocol (Li et al, 2017, *Trials*) |
|  | 4b | Settings and locations where the data were collected. | 5 |
| Interventions | 5 | The intervention and control conditions with sufficient details to allow replication, including whether the intervention was maintained or repeated, and whether it was delivered at the cluster level, the individual participant level, or both. | See published protocol for detailed intervention description (Li et al, 2017, *Trials*) |
| Outcomes | 6a | Completely defined prespecified primary and secondary outcome measures, including how and when they were assessed. | 6; See also published protocol (Li et al, 2017, *Trials*) |
|  | 6b | Any changes to trial outcomes after the trial commenced, with reasons. | n/a |
| Sample size | 7a | How sample size was determined. Method of calculation and relevant parameters with sufficient detail so the calculation can be replicated. Assumptions made about correlations between outcomes of participants from the same cluster. (see separate checklist for SW-CRT sample size items). | 6;  See also published protocol (Li et al, 2017, *Trials*) |
|  | 7b | When applicable, explanation of any interim analyses and stopping guidelines. | n/a |
| Randomisation | | | |
| Sequence generation | 8a | Method used to generate the random allocation to the sequences of treatments. | 7 |
|  | 8b | Type of randomisation; details of any constrained randomisation or stratification, if used. | 7 |
| Allocation concealment mechanism | 9 | Specification that allocation was based on clusters; description of any methods used to conceal the allocation from the clusters until after recruitment. | 7 |
| Implementation | 10a | Who generated the randomisation schedule, who enrolled clusters, and who assigned clusters to sequences. | 7 |
|  | 10b | Mechanism by which individual participants were included in clusters for the purposes of the trial (such as complete enumeration, random sampling; continuous recruitment or ascertainment; or recruitment at a fixed point in time), including who recruited or identified participants. | 7 |
|  | 10c | Whether, from whom and when consent was sought and for what; whether this differed between treatment conditions. | 5 |
| Blinding | 11a | If done, who was blinded after assignment to sequences (eg, cluster level participants, individual level participants, those assessing outcomes) and how. | 7;  See also published protocol (Li et al, 2017, *Trials*) |
|  | 11b | If relevant, description of the similarity of treatments. | n/a |
| Statistical methods | 12a | Statistical methods used to compare treatment conditions for primary and secondary outcomes including how time effects, clustering and repeated measures were taken into account. | 7,8 |
|  | 12b | Methods for additional analyses, such as subgroup analyses, sensitivity analyses, and adjusted analyses. | 7 (See also published protocol (Li et al, 2017, *Trials*)) |
| Results | | | |
| Participant flow (a diagram is strongly recommended) | 13a | For each treatment condition or allocated sequence, the numbers of clusters and participants who were assessed for eligibility, were randomly assigned, received intended treatments, and were analysed for the primary outcome (see separate SW-CRT flow chart). | Figure 2 |
|  | 13b | For each treatment condition or allocated sequence, losses and exclusions for both clusters and participants with reasons. | Figure 2 |
| Recruitment | 14a | Dates defining the steps, initiation of intervention, and deviations from planned dates. Dates defining recruitment and follow-up for participants. | Figure 1-2 |
|  | 14b | Why the trial ended or was stopped. |  |
| Baseline data | 15 | Baseline characteristics for the individual and cluster levels as applicable for each treatment condition or allocated sequence. | Table 2 |
| Numbers analysed | 16 | The number of observations and clusters included in each analysis for each treatment condition and whether the analysis was according to the allocated schedule. | Figure 2 |
| Outcomes and estimation | 17a | For each primary and secondary outcome, results for each treatment condition, and the estimated effect size and its precision (such as 95% confidence interval); any correlations (or covariances) and time effects estimated in the analysis. | 8 |
|  | 17b | For binary outcomes, presentation of both absolute and relative effect sizes is recommended. |  |
| Ancillary analyses | 18 | Results of any other analyses performed, including subgroup analyses and adjusted analyses, distinguishing prespecified from exploratory. | 8-9 |
| Harms | 19 | Important harms or unintended effects in each treatment condition (for specific guidance see CONSORT for harms). | 8 |
| Discussion | | | |
| Limitations | 20 | Trial limitations, addressing sources of potential bias, imprecision, and, if relevant, multiplicity of analyses. | 10-11 |
| Generalisability | 21 | Generalisability (external validity, applicability) of the trial findings. Generalisability to clusters or individual participants, or both (as relevant). | 10 |
| Interpretation | 22 | Interpretation consistent with results, balancing benefits and harms, and considering other relevant evidence. | 10-11 |
| Other information | | | |
| Registration | 23 | Registration number and name of trial registry. | 3 |
| Protocol | 24 | Where the full trial protocol can be accessed, if available. | Li et al, 2017, *Trials* |
| Funding | 25 | Sources of funding and other support (such as supply of drugs), and the role of funders. | 1 |
| Research ethics review | 26 | Whether the study was approved by a research ethics committee, with identification of the review committee(s). Justification for any waiver or modification of informed consent requirements. | 5 |

Table S2: GRIPP2 Checklist for Patient Involvement

| **Aim** | **Report the aim of PPI in the study** |
| --- | --- |
|  | Since the inception of the Canadian Institute of Health Research’s (CIHR) Strategy for Patient Oriented Research (SPOR) in 2011 there has been a marked uptake in partnerships between patients, the public and health research teams.[1, 2] Other countries are also engaging in this new research partnership including the National Institute for Health Research program INVOLVE, in the United Kingdom and the Patient-Centered Outcomes Research Institute (PCORI) in the United States. While a fulsome body of evidence is still emerging on the formal impact this new partnership has had on Canadian health research [3, 4] , many have pursued this novel collaboration based on the moral and political underpinnings of the endeavour.[5–7] Recognising the importance of patient partnerships, the RegisterNow1 Trial team included a patient liaison (LG) with expertise in patient engagement to support training for researchers and citizen partners and to facilitate meaningful contributions from citizen partners. |
| **Methods** | **Provide a clear description of the methods used for PPI in the study** |
|  | **Recruitment**  Word of mouth was used to recruit our citizen partners – members of the study team used social media and personal connections to share the opportunity. Additionally, JM invited MS to the team as they had worked on several organ donation events and campaigns together in the past.  **Training & Orientation**  Recognizing this was the first time both researchers and our citizen partners had been involved in this level of *patient engagement with one another* in a health research context, LG organized a training session and team dinner. The group formally learned about the history and context for patient-oriented research in Canada and began the important work of rapport and trust building while informally gathering in a social setting.  **Defining Terms**  In the training session and through subsequent dialogue the team decided to use the term *citizen partner* rather than the more commonly used term *patient partner*. This was chosen as the participants were not actively seeking medical treatment and saw themselves more as advocates for organ donation.  **Communication** There was regular communication via email and teleconference, in particular in intervention development. The team maintained monthly meetings throughout the grant period and now rely on email to maintain connection. |
| **Study results** | **Outcomes—Report the results of PPI in the study, including both positive and negative outcomes** |
|  | **Results** Our citizen panel was involved in all aspects of the trial from grant application to intervention development (including materials for participants and training for reception staff), interpretation of findings, implementation and knowledge translation.  **Positive Outcomes**  Members of the citizen panel helped to ensure that study materials were written and designed in a manner that people attending a clinic appointment might appreciate, including featuring some of the donor recipient stories of some of our citizen panel members. The PPI in the study also enabled some citizen panel members to become more closely involved in dissemination, including presentations to our partner organ procurement agency,  **Negative Outcomes** There was additional workload to recruit, train and maintain relationships though this was not substantial nor caused a delay for the project. |
| **Discussion and Conclusions** | **Outcomes—Comment on the extent to which PPI influenced the study overall. Describe positive and negative effects** |
|  | During the intervention development phase, the citizen panel reviewed the contents of the pamphlet addressing barriers and enablers to registration that were provided to patients in the trial, with some members featured in the pamphlet itself. They reflected on their experiences visiting their own family physician office to help mitigate potential issues that could arise in delivering the intervention |
| **Reflections/critical perspective** | **Comment critically on the study, reflecting on the things that went well and those that did not, so others can learn from this experience** |
|  | Overall the PPI in this project enabled a range of citizens to actively and meaningfully contribute to multiple phases of the project. The findings from the present study lend themselves to future grant applications for further testing of interventions to increase organ donation registration in family practice. However, post-study communication while waiting for next grant poses some challenges in remaining as connected as we were during the busier project activities. |

References:

1. Demian MN, Lam NN, Mac-Way F, Sapir-Pichhadze R, Fernandez N. Opportunities for Engaging Patients in Kidney Research. Can J Kidney Health Dis. 2017;4:2054358117703070.

2. Government of Canada CI of HR. Strategy for Patient-Oriented Research - Patient Engagement Framework - CIHR. 2014. https://cihr-irsc.gc.ca/e/48413.html. Accessed 3 Aug 2021.

3. Aubin D, Hebert M, Eurich D. The importance of measuring the impact of patient-oriented research. CMAJ. 2019;191:E860–4.

4. Manafo E, Petermann L, Mason-Lai P, Vandall-Walker V. Patient engagement in Canada: a scoping review of the ‘how’ and ‘what’ of patient engagement in health research. Health Research Policy and Systems. 2018;16:5.

5. Esmail L, Moore E, Rein A. Evaluating patient and stakeholder engagement in research: moving from theory to practice. J Comp Eff Res. 2015;4:133–45.

6. Domecq JP, Prutsky G, Elraiyah T, Wang Z, Nabhan M, Shippee N, et al. Patient engagement in research: a systematic review. BMC Health Serv Res. 2014;14:89.

7. Solomon MZ, Gusmano MK, Maschke KJ. The Ethical Imperative And Moral Challenges Of Engaging Patients And The Public With Evidence. Health Aff (Millwood). 2016;35:583–9.

Table S3: Within period and Between Period ICC for donor registration at 1 day, 7 days, 14 days and 30 days

| **Donor Registration Outcome** | **Covariance parameter** | **Estimate** | **Within period ICC** | **Between Period ICC** | **Cluster autocorrelation coefficient** |
| --- | --- | --- | --- | --- | --- |
| 1 day | Intercept | 0.000885 | 0.0042 | 0.0035 | 0.843 |
|  | period | 0.000165 |  |  |  |
|  | Residual | 0.2485 |  |  |  |
| 7 day | Intercept | 0.000911 | 0.0042 | 0.0036 | 0.859 |
|  | period | 0.000149 |  |  |  |
|  | Residual | 0.2486 |  |  |  |
| 14 day | Intercept | 0.000896 | 0.0043 | 0.0036 | 0.843 |
|  | period | 0.000167 |  |  |  |
|  | Residual | 0.2486 |  |  |  |
| 30 day | Intercept | 0.0009 | 0.0043 | 0.0036 | 0.836 |
|  | period | 0.000177 |  |  |  |
|  | Residual | 0.2487 |  |  |  |
| NOTE: from PROC MIXED based on Hooper/Girling model(1, 2) | | | | | |

**References**

1. Girling AJ and Hemming K. Statistical efficiency and optimal design for stepped cluster studies under linear mixed effects models. Stat Med 2016; 35: 2149–2166
2. Hooper R, Teerenstra S, de Hoop E, et al. Sample size calculation for stepped wedge and other longitudinal cluster randomised trials. Stat Med 2016; 35: 4718–4728.

Table S4: Impact of the Intervention on 7-day Donor Registration (primary outcome) by including an interaction between the subgroup variables (male vs. female, age < 40 vs, age ≥ 40) and both the treatment and period indicator

| **Subgroups** | **Total** | **Group 1** | **Group 2** | **Mean (absolute) difference (%)** | | | |  |
| --- | --- | --- | --- | --- | --- | --- | --- | --- |
|  | **24616** | **12132** | **12484** | **Estimate** | **Lower 95%** | **Upper 95%** | **P-value** | **Test for interaction** |
| Male | 8944 | 4436 | 4508 | -0.41% | -4.42% | 3.60% | 0.8027 | 0.9145 |
| Female | 15672 | 7696 | 7976 | -0.61% | -3.92% | 2.71% | 0.6579 |  |
| Age ≤ 40 | 6589 | 3502 | 3087 | 2.21% | -2.37% | 0.07% | 0.2701 | 0.1092 |
| Age > 40 | 18027 | 8630 | 9397 | -1.46% | -4.61% | 0.02% | 0.2858 |  |

NOTE: For the primary outcome of absolute difference (using the identify link) of prevalence registration within 7 days. Confidence interval widths have not been adjusted for multiple testing.
